# Campylobacter jejuni: A Previously Unreported Cause of Toxic Shock Syndrome

**DOI:** 10.7759/cureus.30046

**Published:** 2022-10-07

**Authors:** Glenn E Garcia, Alexander Lake, Domenick Sorresso, Joseph F Staffetti

**Affiliations:** 1 Internal Medicine, HCA Florida Bayonet Point Hospital, Hudson, USA; 2 Gastroenterology, HCA Florida Bayonet Point Hospital, Hudson, USA; 3 Pulmonology and Critical Care, HCA Florida Bayonet Point Hospital, Hudson, USA

**Keywords:** bloody diarrhea, diarrhea, dysentery, gastroenterology, infectious disease, campylobacter jejuni, toxic shock syndrome (tss), campylobacter

## Abstract

This is a case of a previously healthy middle-aged woman who presented with dyspnea after several days of an acute diarrheal illness. She developed acute respiratory distress syndrome (ARDS) requiring mechanical ventilation and met clinical and laboratory criteria for toxic shock syndrome (TSS). Stool studies were positive for *Campylobacter jejuni. *After a literature review, this was found to be the only reported case of *C. jejuni *gastroenteritis leading to TSS. This is the first documentation of TSS as a rare, life-threatening, complication of *Campylobacter* infection.

## Introduction

*Campylobacter jejuni* (*C. jejuni*) is one of the most common bacterial causes of gastroenteritis [1]. This spirochete commonly presents as a self-resolving acute watery diarrhea; however, *C. jejuni* can have life-threatening sequelae such as Guillain-Barré syndrome. We report a case of a 44-year-old African American female who presented with acute hypoxic respiratory failure and was treated for community-acquired pneumonia. She then required mechanical ventilation and developed toxic shock syndrome (TSS) secondary to *C. jejuni*. Physicians must be aware of this never-before-seen sequela of *C. jejuni* infection, as it may present in other infections previously unassociated with TSS.

This case was accepted for poster presentation at the American College of Gastroenterology 2022 Annual Scientific Meeting on October 24, 2022. 

## Case presentation

A previously healthy 44-year-old African American female presented to our hospital with a chief complaint of shortness of breath, associated with cough and weakness of three days duration. She reported having five to six episodes daily of bloody diarrhea for several days prior to the onset of her respiratory symptoms. She denied taking any medications at home. The patient works as a nursing assistant at a local nursing home. She denied recent travel and any sick contacts but acknowledged that she does not know the medical history of the patients at her facility. Her mother has a history of type 1 diabetes mellitus; there is no family history of inflammatory bowel disease.

On admission, the patient had a temperature of 38.0°F, a heart rate of 136 beats per minute, 22 respirations per minute, oxygen saturation of 94% on room air, and blood pressure of 117/71. Physical examination revealed tachypnea and diffuse abdominal tenderness and guarding. The lungs were clear to auscultation bilaterally. Chest x-ray showed no acute cardiopulmonary disease. Electrocardiogram was read as atrial fibrillation with a rapid ventricular response. Computerized tomography (CT) angiography of the chest ruled out pulmonary emboli but showed a possible right lower lobe pneumonia. CT of the abdomen and pelvis was normal except for mild splenomegaly. Significant laboratory values are seen in Table 1. The patient was started on intravenous (IV) fluids and treated empirically with ceftriaxone 1g IV daily and azithromycin 500mg IV daily for community-acquired pneumonia. 

**Table 1:** Notable Laboratory Findings AST: aspartate aminotransferase; ALT: alanine transaminase

Test	Reference Range	Day 1	Day 4	Day 7	Day 10
Hemoglobin	12.0 - 18.0 g/dl	8.6 g/dL	6.8 g/dL	8.9 g/dL	9.3 g/dL
White Blood Cell Count	4 -10.5 x 10^3^cells/μL	10.6 x 10^3^cells/μL	20.1 x 10^3^ cells/μL	52.6 x 10^3^cells/μL	28.5 x 10^3^cells/μL
Platelets	150-450 x 10^3^ PLT/μL	196 x 10^3^PLT/μL	74 x 10^3^PLT/μL	81 x 10^3^PLT/μL	73 x 10^3^PLT/μL
Total Bilirubin	0.20-1.00 mg/dL	0.95 mg/dL	2.26 mg/dL	4.07 mg/dL	8.62 mg/dL
Direct Bilirubin	0.0-0.20 mg/dL	0.44 mg/dL	N/A	2.95 mg/dL	6.56 mg/dL
AST	15-37 Units/L	168 U/L	68 U/L	85 U/L	41 U/L
ALT	12-78 Units/L	128 U/L	51 U/L	42 U/L	50 U/L

On the fourth day, the patient’s leukocytosis worsened, and her temperature increased to 40.5°C. The patient received one unit of packed red blood cells for hemoglobin of 6.8 g/dL. Later that day, the patient had increased oxygen requirements going from a nasal cannula to a nonrebreather mask with fraction of inspired oxygen (FiO2) of 100%. On physical examination, the patient exhibited an erythematous tongue and white lesions on the oropharyngeal mucosa, bilateral crackles, and a diffuse, confluent orange rash that involved the palms. She was now encephalopathic. Right upper quadrant ultrasound with Doppler did not show any liver lesions or a portal thrombus. Fluconazole 200mg IV daily was started for oral thrush. That night, the patient continued to decompensate and became hypotensive (mean arterial pressure of 58) despite being bolused 6L of normal saline. She was transferred to the ICU for septic shock, intubated, and placed on mechanical ventilation. Initial ventilator settings were assist-control (AC) mode, respiratory rate of 18, tidal volume of 500mL, and positive end-expiratory pressure of 5 cmH_2_O. Upon admission to the ICU, antimicrobial coverage was broadened to include vancomycin 1.5g IV daily and cefepime 2g every eight hours. Blood and sputum cultures showed no growth. 

By the seventh day, the patient's condition worsened. Her fevers fluctuated between 38-39˚C, and the white blood cell count increased to 52.6 x 10^3^ cells/mm3. Her maximum temperature was 40.5˚C on day four. She developed bullae on her extremities and in the gluteal cleft, which exhibited the characteristic Nikolsky sign. Due to skin sloughing, a rectal tube was inserted, and stool samples were sent for culture. The differential diagnosis now expanded to include TSS and Stevens-Johnson syndrome. There was sloughing of the vaginal mucosa on pelvic examination, but no foreign bodies. Fluconazole was discontinued, as the oral lesions were now attributed to mucosal sloughing. The patient was started on clindamycin 900mg IV every eight hours.

The patient's vasopressors (phenylephrine and vasopressin) were titrated over the next few days. Stool studies were positive for *C. jejuni*. Skin biopsies and immunofluorescence were not consistent with Stevens-Johnson syndrome. The diagnosis of TSS was made. The Infectious Disease consultant ordered a lumbar puncture, which was suspicious for meningitis (Table 2); however, the CSF was not cultured in a growth medium conducive to *C. jejuni*. The patient was started on meropenem 2g IV every eight hours as empiric treatment for *Campylobacter *meningitis; treatment was continued for four weeks due to the severity of the patient's illness. After a long hospital course complicated by critical illness myopathy, the patient was stabilized and discharged to a rehabilitation facility. After one month, she made a full recovery and returned to work.

**Table 2:** Lumbar Puncture Results PMNs: polymorphonuclear neutrophils

Test	Patient Results
Fluid Color	Pale Red
Fluid Appearance	Cloudy
Fluid WBC	92 cells/µL (92% PMNs)
Fluid RBC	18000 cells/µL
CSF Glucose	99 mg/dL
CSF Protein	35.4 mg/dL
Oligoclonal Bands	0

## Discussion

TSS is a toxin-mediated, multi-system inflammatory condition. It is classically known in the context of a staphylococcal or streptococcal infection and in retained menstrual products (e.g., tampons). The toxins produced in these settings act as superantigens, which induce cytokines to trigger an inflammatory cascade. An equivalent to the TSS toxin (TSST-1) produced by *Staphylococcus aureus* has not been identified in *Campylobacter *species [2]. With all cultures negative for *S. aureus*, it cannot be a cause of our patient's TSS. Thus, we submit that an alternative route with a toxin produced by *Campylobacter *species has led to this case of TSS. As cytotoxins (as opposed to enterotoxins) are implicated in the pathogenesis of bloody diarrhea [3], *Campylobacter *cytotoxin is known to be immunogenic and is suspected to have mediated our patient's inflammatory response [2].

Our patient exhibited fever (40.5°C), diffuse erythroderma, skin desquamation, hypotension, and a constellation of gastrointestinal, mucosal, hepatic, and hematologic involvement. Thus, we are confident in the diagnosis of TSS (Table 3). The medical literature has only reported one case of campylobacteriosis leading to TSS, caused by *Campylobacter intestinalis*. [4] There is debate as to whether *C. jejuni *makes an exotoxin capable of this level of inflammatory response [5-7]. This appears to be the only reported case of *C. jejuni* causing TSS. Blood, sputum, stool, and urine cultures did not yield other results and a skin biopsy ruled out Stevens-Johnson syndrome. Therefore, we are confident our patient had TSS due to *C. jejuni*.

**Table 3:** Criteria for Diagnosis of Toxic Shock Syndrome Based on: CDC's Case Definition of Toxic Shock Syndrome (Other Than Streptococcal) (TSS)) [8] ALT: alanine aminotransferase; AST: aspartate aminotransferase; BUN: blood urea nitrogen

Classification		
	Probable: Case meets laboratory criteria and 4/5 clinical criteria.	
	Confirmed: Case meets laboratory criteria and 5/5 clinical criteria - including desquamating rash.	
Laboratory Criteria		
	Negative blood or cerebrospinal fluid cultures; may be positive for* Staphylococcus aureus*	
	Negative serologies for leptospirosis, measles, or Rocky Mountain spotted fever	
Clinical Criteria		
	Temperature ≥ 38.9˚C (102.0˚F)	
	Diffuse macular erythroderma	
	Desquamating rash	
	Systolic blood pressure ≤ 90 mmHg	
	Multisystem Involvement (≥ 3)	
		Central Nervous System: Encephalopathy in the absence of fever and hypotension
		Gastrointestinal: Nausea, vomiting
		Hematologic: Platelets less than 100,000 per cubic mm
		Hepatic: Total bilirubin, ALT, or AST > 2x upper limit of normal
		Musculoskeletal: Creatine phosphokinase > 2x upper limit of normal or severe myalgia
		Mucosal: Conjunctival, oral, or vaginal hyperemia
		Renal: BUN or creatinine > 2x upper limit of normal or sterile pyuria

## Conclusions

This is a unique presentation of *C. jejuni *infection leading to TSS. This case highlights the importance of stool cultures and assays in acute diarrheal illnesses. Clinicians must also be willing to expand the differential diagnoses to include uncommon or (as in our case) unreported complications of common diseases. Further research is needed to identify the immunogenic toxins produced by this organism and to determine a mechanism for the systemic inflammatory response seen in our patient.
